# Advancements in skeletal muscle tissue engineering: strategies for repair and regeneration of skeletal muscle beyond self-repair

**DOI:** 10.1093/rb/rbaf050

**Published:** 2025-05-28

**Authors:** Lei Qi, Fengyuan Zhang, Kexin Wang, Bingqian Chen, Xia Li, Jin Xu, Jiacheng Sun, Boya Liu, Zihui Gao, Yanan Ji, Leilei Gong, Youhua Wang, Xinlei Yao, Xiaosong Gu, Hualin Sun

**Affiliations:** Key Laboratory of Neuroregeneration of Jiangsu and Ministry of Education, Affiliated Hospital of Nantong University, Medical School of Nantong University, Co-Innovation Center of Neuroregeneration, Nantong University, Nantong, Jiangsu Province 226001, People’s Republic of China; Department of Emergency Medicine, Affiliated Hospital of Nantong University, Nantong, Jiangsu Province 226001, People’s Republic of China; Key Laboratory of Neuroregeneration of Jiangsu and Ministry of Education, Affiliated Hospital of Nantong University, Medical School of Nantong University, Co-Innovation Center of Neuroregeneration, Nantong University, Nantong, Jiangsu Province 226001, People’s Republic of China; Key Laboratory of Neuroregeneration of Jiangsu and Ministry of Education, Affiliated Hospital of Nantong University, Medical School of Nantong University, Co-Innovation Center of Neuroregeneration, Nantong University, Nantong, Jiangsu Province 226001, People’s Republic of China; Department of Orthopedics, Changshu Hospital Affiliated to Soochow University, First People’s Hospital of Changshu City, Changshu, Jiangsu Province 215500, People’s Republic of China; Key Laboratory of Neuroregeneration of Jiangsu and Ministry of Education, Affiliated Hospital of Nantong University, Medical School of Nantong University, Co-Innovation Center of Neuroregeneration, Nantong University, Nantong, Jiangsu Province 226001, People’s Republic of China; Departmentof Basic Medicine, Kangda College of Nanjing Medical University, Lianyungang, Jiangsu Province 222000, People’s Republic of China; Key Laboratory of Neuroregeneration of Jiangsu and Ministry of Education, Affiliated Hospital of Nantong University, Medical School of Nantong University, Co-Innovation Center of Neuroregeneration, Nantong University, Nantong, Jiangsu Province 226001, People’s Republic of China; Key Laboratory of Neuroregeneration of Jiangsu and Ministry of Education, Affiliated Hospital of Nantong University, Medical School of Nantong University, Co-Innovation Center of Neuroregeneration, Nantong University, Nantong, Jiangsu Province 226001, People’s Republic of China; Key Laboratory of Neuroregeneration of Jiangsu and Ministry of Education, Affiliated Hospital of Nantong University, Medical School of Nantong University, Co-Innovation Center of Neuroregeneration, Nantong University, Nantong, Jiangsu Province 226001, People’s Republic of China; Key Laboratory of Neuroregeneration of Jiangsu and Ministry of Education, Affiliated Hospital of Nantong University, Medical School of Nantong University, Co-Innovation Center of Neuroregeneration, Nantong University, Nantong, Jiangsu Province 226001, People’s Republic of China; Key Laboratory of Neuroregeneration of Jiangsu and Ministry of Education, Affiliated Hospital of Nantong University, Medical School of Nantong University, Co-Innovation Center of Neuroregeneration, Nantong University, Nantong, Jiangsu Province 226001, People’s Republic of China; Research and Development Center for E-Learning, Ministry of Education, Beijing 100816, People’s Republic of China; Key Laboratory of Neuroregeneration of Jiangsu and Ministry of Education, Affiliated Hospital of Nantong University, Medical School of Nantong University, Co-Innovation Center of Neuroregeneration, Nantong University, Nantong, Jiangsu Province 226001, People’s Republic of China; Key Laboratory of Neuroregeneration of Jiangsu and Ministry of Education, Affiliated Hospital of Nantong University, Medical School of Nantong University, Co-Innovation Center of Neuroregeneration, Nantong University, Nantong, Jiangsu Province 226001, People’s Republic of China; Key Laboratory of Neuroregeneration of Jiangsu and Ministry of Education, Affiliated Hospital of Nantong University, Medical School of Nantong University, Co-Innovation Center of Neuroregeneration, Nantong University, Nantong, Jiangsu Province 226001, People’s Republic of China; Key Laboratory of Neuroregeneration of Jiangsu and Ministry of Education, Affiliated Hospital of Nantong University, Medical School of Nantong University, Co-Innovation Center of Neuroregeneration, Nantong University, Nantong, Jiangsu Province 226001, People’s Republic of China

**Keywords:** skeletal muscle tissue engineering, seed cells, biomaterials, advanced techniques

## Abstract

Skeletal muscle is a vital organ of exercise and energy metabolism, playing a crucial role in maintaining body posture, enabling movement and supporting overall health. When skeletal muscle undergoes minor injuries, it has the inherent ability to self-repair and regain function. However, the ability of skeletal muscle self-repair is affected in severe muscle damage, resulting in significant muscle loss and functional impairments. For the severe muscle injury, tissue engineering strategies are used as the new methods to promote the repair and regeneration of skeletal muscle. Skeletal muscle tissue engineering (SMTE) aims to repair or regenerate skeletal muscle using seed cells, scaffolds, bioactive molecules or their combinations to reverse muscle loss caused by traumatic injury or congenital muscle defects. In this study, we provide an overview of the structure and contraction process of skeletal muscle, as well as its mechanisms of natural repair and regeneration. We describe the seed cells with myogenic potential and show natural, synthetic and composite biomaterials, as well as advanced technologies for manufacturing scaffolds used in SMTE. SMTE has broad prospects, but it still faces many challenges before clinical application. The continued advancement of muscle tissue engineering will yield innovative outcomes with significant clinical potential for skeletal muscle regeneration.

## Graphical abstract

**Figure:**
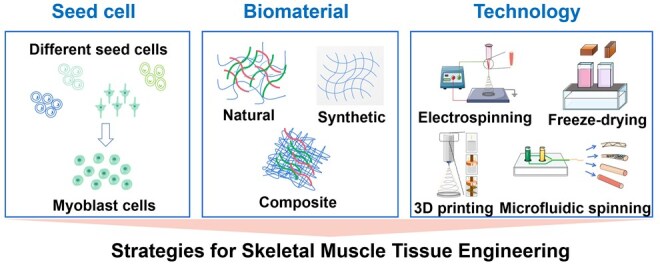

## Introduction

Skeletal muscle is an integral part of the body, accounting for more than 40% of the body mass. It is a highly adaptable organ regulated by various cellular and molecular mechanisms [1–4]. This organ plays a vital role in many dynamic functions of the body and maintains internal balance, including force generation, chewing, body posture maintenance, organ protection, movement, respiration and thermogenesis [3, 5–7]. Moreover, it also secretes myokines, metabolites, microRNAs, exosomes and other factors to participate in the regulation of systemic metabolism [2]. Skeletal muscle is a pivotal organ of exercise and energy metabolism. Preserving its homeostasis is critical to sustaining systemic physiological functions.

A notable characteristic of skeletal muscle is its limited ability to coordinate repair and regeneration following injury. Its regeneration depends on the muscle stem cells, known as satellite cells (SCs) [8]. SCs are a population of progenitor cells, which are usually located between muscle fibers or within the basal lamina. They can proliferate and differentiate, thereby promoting muscle growth and regeneration, providing new nuclei for muscle fibers to increase and maintain muscle quality, and ensuring the maintenance, growth and repair of skeletal muscle [9]. Under normal conditions, SCs remain quiescent; however, they become activated and expand in response to injury or stress. On the one hand, they maintain a viable pool of SCs through self-renewal; on the other hand, they generate myoblasts that proliferate and subsequently differentiate into multinucleated myotubes, either forming *de novo* muscle fibers or fusing with pre-existing damaged fibers to mediate tissue regeneration [10, 11]. Skeletal muscle possesses the inherent ability to self-repair and maintain tissue integrity after minor injuries (such as strains, tears and contusions) [12]. However, severe defects or injuries, such as volumetric muscle loss (VML) from trauma or surgery, chronic and incomplete healing due to genetic muscle disorders and age-related sarcopenia, result in substantial loss of stem cells and extracellular matrix (ECM), leading to chronic inflammation and fibrosis. Consequently, the regenerative ability of SCs diminishes, hindering muscle regeneration and causing functional decline in the affected muscle, thereby impairing both muscle and overall health to varying degrees [13–15].

At present, diverse therapeutic approaches have been developed to enhance skeletal muscle regeneration, including surgical interventions, pharmacological treatments, physical therapies, cell-based therapies, nanotechnologies and tissue engineering [16, 17]. Tissue engineering converges principles from materials science, cell biology and engineering to seek to regenerate tissues and organs. Because of its potential to regenerate tissues, tissue engineering is considered to be a meaningful method of tissue regeneration [18, 19]. In general, tissue engineering uses seed cells combined with suitable biomaterials to produce a favorable microenvironment for functional repair, replacement and regeneration of damaged organs [10, 20]. Given the limitations of intrinsic tissue regeneration, tissue engineering offers a viable alternative for restoring damaged muscle tissue. Tissue engineering has three basic elements: seed cells that proliferate and differentiate into tissues and organs, growth factors that regulate cell proliferation and differentiation and biomaterials that support cell proliferation and differentiation [19, 21, 22]. Skeletal muscle tissue engineering (SMTE) employs myogenous cells, scaffolds, bioactive molecules or their combinations to reverse volumetric muscle loss resulting from severe trauma or congenital abnormalities and support the formation of skeletal muscle tissue [23]. SMTE can be categorized into three main approaches: *in situ*, *in vivo* and *in vitro*. *In situ* SMTE utilizes the natural regenerative potential to achieve tissue repair by implanting cell-free biomaterial scaffolds at the injury site. The biomaterials can release biophysical and biochemical signals to guide endogenous cells to the injured site and induce endogenous tissue regeneration by regulating the extracellular microenvironment or driving cell reprogramming [24, 25]. *In vivo* SMTE depends on the transplantation of muscle-derived cells, which are first seeded onto a guiding biomaterial scaffold before being implanted into the body to participate in regeneration. The cells are susceptible to low viability and immune rejection. Whether alone or combined with a scaffold/scaffolds, it is necessary to reconstruct the local microenvironment and allow cells to integrate or promote the formation of new tissues [26]. *In vitro* SMTE involves the implantation and development of constructs, which are achieved by combining biological factors, scaffolds and cells, followed by an extensive *in vitro* cultivation period, until the cells differentiate into contractile muscle fibers, so as to enable transplantation into patients [27, 28]. With the further research on tissue engineering of skeletal muscle, it is expected that more and more products will be used for repairing skeletal muscle. Tissue engineering has broad prospects in skeletal muscle regeneration, but it still faces many challenges before clinical application. The continued evolution of SMTE promises to establish a robust translational bridge between engineered skeletal muscle constructs and clinically viable solutions for functional restoration, ultimately enabling intervention against volumetric muscle loss.

## Composition of skeletal muscle

**Figure 1.**
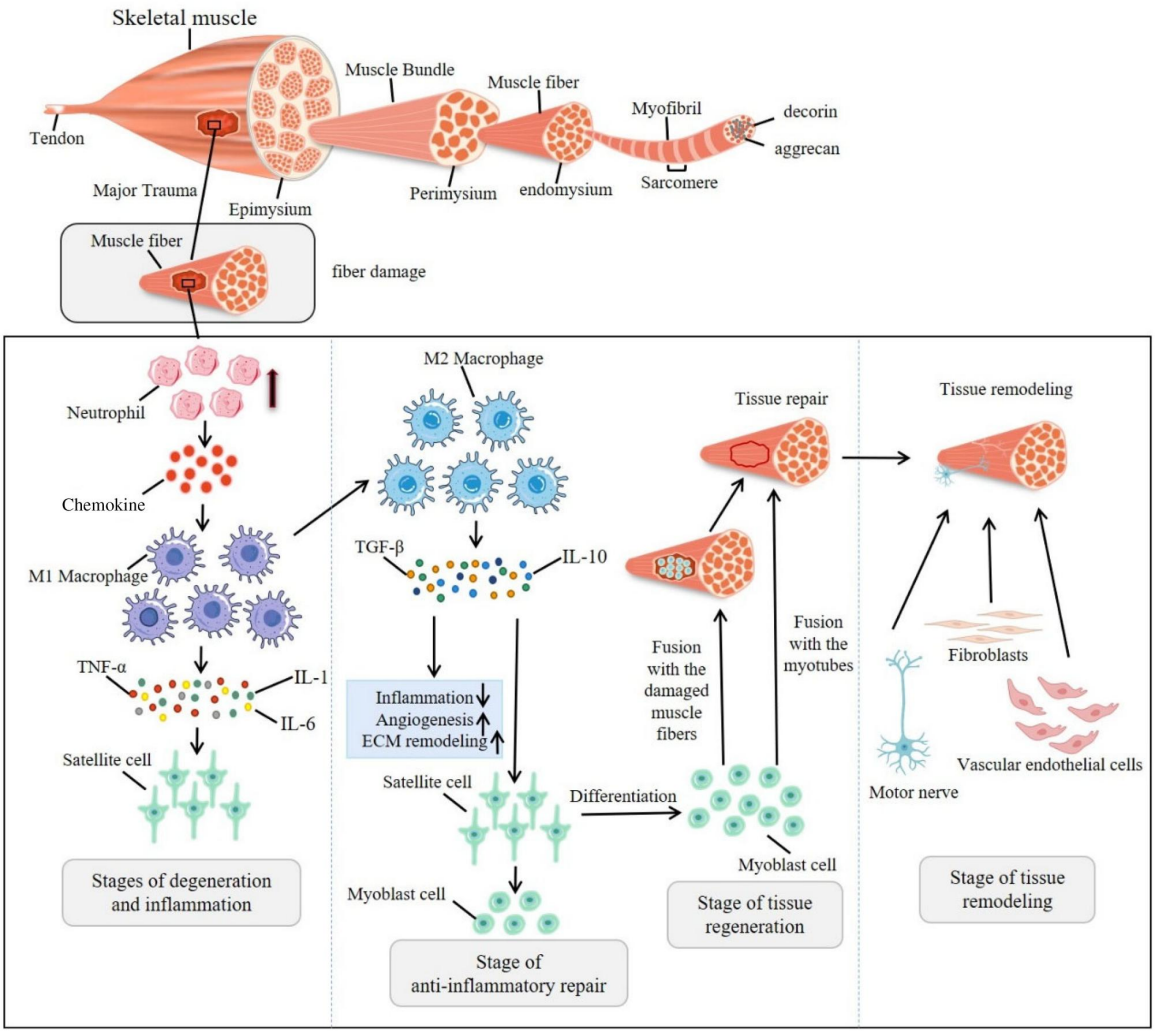
The structure, repair, regeneration and remodeling of skeletal muscle.

Skeletal muscle is a dynamic organ comprised of contractile muscle fibers, blood vessels, connective tissue and nerve endings [29]. It is fixed to the bone through tendons, which is composed of bundles of muscle fibers containing thousands of myofibrils. The surface of skeletal muscle is covered with epimysium formed by dense connective tissue, which separates it from surrounding tissues [30]. In the cross-sections of skeletal muscle, the outer epimysium and inter different numbers of muscle bundles can be observed. The muscle bundle is ensheathed by a thin layer of loose connective tissue called the perimysium, which binds muscle fibers together to form bundles. The thin layer of connective tissue coated around each muscle fiber is endomysium, which is rich in capillaries, neuronal axons and muscle stem cells (MuSCs) [31, 32] (Figure 1). Skeletal muscle fibers are multinucleate cells formed by fusion of myocytes. The cells vary in length, are closely arranged and complementary [33, 34]. Muscle fibers are also surrounded by a specialized ECM, which is composed of reticular lamina and basal lamina, and MuSCs. The core of the basal lamina is composed of self-assembled polymeric laminin and collagen network. The collagen network binds proteoglycan, provides transverse cross-linking, establishes indirect contact with cell surface receptors, and serves as a reservoir of growth factors [35]. Together, these components form the fundamental structure of skeletal muscle. As a complex muscle connective tissue, it is composed of noncontractile ECM embedded in fibroblasts and macrophages, along with a dense network of capillaries and neural connections. It has enough flexibility to adapt to contraction/relaxation cycle and determine tissue elasticity [36]. The endomysium, epimysium and perimysium together provide structural support for the muscle tissue, and contribute to synchronous contraction and force transmission. Skeletal muscle is also highly vascularized and innervated, connected to the vascular network to receive nutrients and remove metabolites, and connected to the neural network to activate and contract [37]. Maintaining the normal function of skeletal muscle is essential for good health.

Within the complex microenvironment of skeletal muscle, there are multiple types of progenitor cells and stem cells that work together to maintain skeletal muscle homeostasis. Among them, the most important muscle stem cells are MuSCs, which can differentiate into myoblasts and promote muscle regeneration upon exposure to external stimuli [38]. Pax7 is a marker of MuSCs. PW1^+^/Pax7^-^ mesenchymal progenitor cells (PICs) exhibit myogenic capacities, contributing to skeletal muscle regeneration. They also secrete a series of growth factors that promote muscle repair, forming a favorable microenvironment, thereby enhancing muscle regeneration [8, 39]. In addition, a group of pericytes around blood vessels in skeletal muscle not only cooperate with endothelial cells to participate in vascular stability and maturation but also demonstrate differentiation capacity into myogenic lineages and contribute to postinjury muscle regeneration [40]. Side population cells are another type of myogenic progenitor cells that rapidly proliferate after muscle injury and exhibit high myogenic potential [8]. Similarly, CD133^+^ cells are a type of pluripotent stem cell derived from peripheral blood cells, expressing myogenic markers in the basal lamina of muscle fibers and possessing myogenic potential [8, 41]. In addition, Twist2^-^ cells are predominantly located in the muscle interstitium, leading to the formation of specific fiber types, playing an indispensable role in muscle formation [42]. The fibro-adipogenic progenitors (FAPs) can rapidly proliferate and differentiate into adipocytes or fibroblasts during muscle injury, helping to repair damage of skeletal muscle [43]. In skeletal muscle, there are also some endothelial cells that form the inner walls of blood vessels within the muscle and participate in muscle homeostasis regulation together with surrounding cells [44]. These cells together form the complex cell network of skeletal muscle, ensuring its normal function and ability to respond to injury.

Myosin and actin are bound to myofibrils, and their interaction is the basis for contraction and force generation of muscle fibers [45]. Myofibrils exhibit light and dark bands. The dark band is called the A band, which contains a narrower band called the H band. In the center of the H band, there is a dark linear structure called M line. The light band is I band, and there is a dark linear structure called Z line in the center of I band [36]. Myofibrils alternate between bright bands and dark bands, repeatedly. A section of myofibril between two adjacent Z lines is sarcomere, which is the basic unit of structure and function of myofibril, and is also the basic contraction unit of skeletal muscle [46] (Figure 1). The length of sarcomere changes with the contraction and relaxation of muscle fibers. In addition, sarcomere is composed of regularly arranged thick myofilaments and thin myofilaments. The thick myofilaments are formed by hundreds of myosin molecules arranged in bundles, while the thin myofilaments are a complex of actin, tropomyosin and troponin [47]. Muscle fiber contraction is accomplished by sliding the thin myofilament to the thick myofilament within the sarcomere [48]. The muscle stimulation leads to increased calcium concentrations in muscle fibers, and calcium ions bind to troponin, resulting in tropomyosin movement. The binding part of myosin and actin is exposed. Myosin binds to actin, ADP and phosphate are released, and myosin bends and pushes actin. Then, ATP binds to myosin, causing it to release actin; ATP is hydrolyzed into ADP and phosphate, and myosin binds to actin again [36]. This process will continue when the calcium concentration is high in muscle fibers. When the stimulation ends, the calcium concentration decreases, and the complex of troponin and tropomyosin returns to the static state [49, 50]. The normal contraction and relaxation of muscle is the key to the body to achieve movement and maintain posture.

Mechanical output in skeletal muscle is determined by both cross-sectional dimensions and phenotypic heterogeneity of myofibers, with type I (slow-twitch) and type II (fast-twitch), including subtypes of IIA, IIB and IIx [51]. Type I fiber shows red due to its myoglobin content. They have a slow contraction speed, low contraction force, high contents of mitochondria and oxidase and generate ATP through oxidative reactions, making them resistant to fatigue. Type II fiber shows white owing to the lack of myoglobin, and generates ATP through glycolysis. It has large contraction force and is liable to fatigue [50, 52]. Under pathological conditions, the fiber type of skeletal muscle will change, which may aggravate the damage to the normal function of skeletal muscle. The current research has found the changes of fiber type in muscle atrophy caused by diabetes [53, 54]. In addition, chronic inactivity and muscle disorder are also considered to cause fiber type conversion of slow to fast in lower limb muscles during chronic disease [55]. Evidence suggests that an elevated proportion of type I myofibers composition is associated with therapeutic benefits for sarcopenia attenuation and metabolic disorder mitigation [56]. The transformation from fast fiber to slow fiber in muscle is of great significance for continuous contraction and extension events, energy homeostasis and anti-fatigue ability [57]. This provides a new direction for improving damage and maintaining homeostasis in skeletal muscle.

## Repair and regeneration of skeletal muscle

The injury of muscle is a common clinical condition, which can be divided into acute injury (such as fracture, cut, sprain and contusion) and chronic injury (such as ischemic injury and muscle atrophy disease). The repair and regeneration of injured skeletal muscle includes three overlapping phases: degeneration and inflammation, anti-inflammatory repair and tissue remodeling.

### Degeneration and inflammation

The degenerative and inflammatory phases begin within a few minutes after muscle injury and last for 1–2 weeks. At this phase, the damage of sarcolemma and basal lamina caused by injury leads to the influx of extracellular calcium, resulting in the self-digestion and necrosis of muscle fibers, followed by the infiltration of immune cells [58]. At the beginning of the injury, neutrophils are the first immune cells to infiltrate the injury site. They increase in number, secrete chemokines, recruit macrophages to the injury site and promote the inflammatory response [59, 60]. Macrophages play dual roles in skeletal muscle regeneration, exhibiting both immune and non-immune functions. They show nutritional roles in muscle cells and mesenchymal stem cells and produce inflammatory reactions [61]. Proinflammatory (M1) macrophages, which express CD86, clear necrotic cells and fragments from the injured area, and secrete various proinflammatory factors, including IL-6, IL-1β and TNF-α, and stimulate the proliferation of MuSCs [62, 63] (Figure 1). The pro-inflammatory phase is usually 2–3 days. Although inflammation is often viewed as detrimental, it is essential for the recovery of muscle tissue after injury.

### Anti-inflammatory repair

The anti-inflammatory repair phase begins 2–3 days post-injury in muscle tissue. The pro-inflammatory macrophages transform into anti-inflammatory (M2) macrophages, triggering the disappearance of inflammation. The mitogen-activated protein kinase 1 (MKP-1) and adenosine monophosphate activated protein kinase (AMPK) pathways are required for macrophage transition during muscle regeneration. MKP-1 inhibits the activation of p38 MAPK, thereby preventing Akt activation and enabling the transformation of M1 macrophages into M2 macrophages [64]. In addition, regulatory T lymphocytes also promote and regulate the transition, and can activate proliferation and differentiation of SCs [44]. Anti-inflammatory macrophages, which express arginase 1, CD163 and CD206, secrete IL-10 and TGF-β and promote the repairation and regeneration of damaged muscle [61, 65]. Macrophages can phagocytize dead cells and cell fragments through endocytosis, which is also necessary for the elimination of inflammation [66]. For example, when macrophages phagocytize dead myoblasts, they cease the secretion of TNF-α and anti-inflammatory effectors such as TGF-β [67]. During the recovery phase, macrophages inhibit inflammation, promote stem cell differentiation, angiogenesis and ECM remodeling by secreting proteoglycans, matrix cell proteins and assemble proteins [61, 67]. The phenotypic shift of macrophages from inflammatory to anti-inflammatory state plays a vital role in skeletal muscle injury recovery.

During the regenerative phase, MuSCs play an integral role in muscle repair and regeneration. Studies have demonstrated that muscle regeneration fails when Pax7^+^ satellite cells were depleted, indicating that no other cell types can compensate for the myogenic function of Pax7^+^ cells during injury [68]. In the steady state, SCs remain quiescent and are located between the basal lamina and sarcolemma [32]. During the inflammatory phase, these quiescent SCs are activated by growth factors and cytokines released at the site of injury, prompting them to re-enter the cell cycle and differentiate into myoblasts [63] (Figure 1). MuSCs are activated and proliferated by specific transcription factors, including Pax7, MyoD, myogenin and MEF2C, and then, myogenically differentiated. Pax7 is crucial for maintaining SC quiescence and self-regeneration, while MyoD and Myf5 drive the early myogenic fate, and myogenin is crucial for differentiation. MRF4 is involved in the maturation of myotubes during the late phase of regeneration [44]. Differentiated myoblasts either fuse with damaged muscle fibers to facilitate repair or undergo proliferation, differentiation and fusion with myotubes to produce new muscle fibers [69–71] (Figure 1). The differentiation is accompanied by the dynamic regulation of key transcription factors, involving both downregulation and upregulation. A small portion of SCs retains a quiescent state to replenish the stem cell pool, ensuring the long-term maintenance of muscle regenerative capacity [72]. Proliferating myoblasts exhibit bidirectional migration toward the injury site, guided by residual ECM of damaged muscle fibers and chemotactic gradients produced by infiltrating macrophages and damaged myofibers [32]. This process relies on dynamic cytoskeletal rearrangements.

### Tissue remodeling

Tissue remodeling begins 2–3 weeks after injury. During the remodeling phase, damaged connective tissue undergoes repair through fibroblast infiltration into the wound microenvironment. However, excessive ECM production from fibroblasts may reshape connective tissue into scar tissue, damaging muscle fiber contractility and tissue structure in larger injuries [73, 74]. Vascular endothelial cells proliferate and migrate to the surrounding tissues, contributing to the generation of new blood vessels in the injured muscle. Finally, the motor nerve reinnervates the nascent muscle fibers to form new neuromuscular junctions [32]. Following completion of regeneration, the majority of inflammatory cells either retreat from the injury area or undergo programmed cell death, thereby reestablishing tissue equilibrium.

The muscle is composed of muscle bundles, each containing numerous muscle fibers. Each myofiber is ensheathed within the endomysium with clustered fibers forming fascicles through perimysial organization, ultimately being circumscribed by the epimysium as the muscle's outer envelope. The myofibrils within the muscle fibers contain sarcomeres, the basic contractile units of muscle. The tendon, which attaches the muscle to the bone, is composed of collagen and other ECM proteins such as decorin and aggrecan. Muscle Injury and Inflammatory Response: Major trauma leads to muscle fiber damage, triggering an inflammatory response. Neutrophils are the first responders, secreting chemokines that attract M1 macrophages. These M1 macrophages secrete inflammatory mediators including IL-1, TNF-α and IL-6, which further promote inflammation and satellite cell activation. Anti-Inflammatory Repair: In this stage, M2 macrophages are recruited, which secrete anti-inflammatory mediators including TGF-β and IL-10. These mediators promote angiogenesis and ECM remodeling, facilitating the transition from inflammation to repair. Satellite cells, under the influence of these cytokines, differentiate into myoblasts. Myoblasts integrate into damaged muscle fibers, contributing to tissue repair, or they undergo mutual fusion to generate new myotubes. Tissue Remodeling: The process involves the activation of fibroblasts and vascular endothelial cells, which are vital to tissue remodeling. The motor nerve also contributes to the regeneration process by providing necessary signals for muscle cell differentiation and fusion.

## Seed cells for skeletal muscle tissue engineering

Seed cells are crucial for tissue regeneration, with sources comprising autologous, allogeneic and xenogeneic cells. Autologous cells are extracted from the patient's own tissues, which eliminate the risk of immune response, but have limited sources [75]. Autologous therapy is only applicable to patients with genetically healthy muscles. For muscular dystrophies such as Duchenne muscular dystrophy (DMD), autologous therapy is not an option unless the patient's cells experience genetic modification [76]. Allogeneic cells are provided by donors, which are not affected by the patient's own body conditions and have a higher survival rate, but donors are limited, expensive and have a high probability of immune rejection [77]. Xenogeneic cells are obtained from animals, which are common sources of human tissue engineering and regenerative medicine applications, but they present issues such as immune rejection, viral contamination, ethics and other problems [78]. Nowadays, a major challenge in skeletal muscle regenerative medicine lies in identifying a suitable stem cell source to achieve extensive cell expansion and avoid losing myogenic potential (Table 1) [79].

### Satellite cells

SCs are the most thoroughly researched cell type for this application because they are the natural precursors of muscle tissue and can differentiate and produce new muscle cells, which is considered to be the ideal cell type for muscle tissue development *in vitro* [80]. SCs play a key role in repairation and regeneration in skeletal muscle. When muscle fibers damage, quiescent SCs become activated, proliferate and produce muscle derived progenitor cells (MPC). MPCs differentiate into myoblasts, which are capable of fusing to create multinucleated myotubes. Eventually, these myotubes develop into new muscle fibers [81]. In fact, although SCs are effective muscle stem cells, they have high cost of enzymatic hydrolysis, low separation rate, complex purification procedures, and their proliferation ability is limited *in vitro* [14]. These limitations of SCs prompted researchers to study other stem cell populations to regenerate skeletal muscle tissue.

### Mesenchymal stem cells

Mesenchymal stem cells (MSCs) are a type of adult stem cells, which have shown the ability of myogenic differentiation. MSCs have the ability of easy separation, *ex vivo* expansion, self-renewal, pluripotency, immune regulation and the secretion of growth factors and cytokines, showing excellent candidates for use in tissue engineering [82, 83]. Bone marrow (BM) and adipose tissue are the primary sources of MSCs [81]. Bone marrow mesenchymal stem cells (BM-MSCs) have myogenic, neurogenic and vascular differentiation ability, which may contribute to tissue repair [84]. First, BM-MSCs secrete brain-derived neurotrophic factor (BDNF), vascular endothelial growth factor (VEGF), insulin-like growth factor-1 (IGF-1), sphingosine 1-phosphate (S1P) via paracrine to promote the proliferation and differentiation of myoblasts and SCs, and regulate skeletal muscle regeneration. Among them, VEGF derived from BM-MSCs has been proved to upregulate mTOR expression through JAK/STAT3 or PI3K/AKT signaling pathways and promote the differentiation of myoblasts [84, 85]. In addition, BM-MSCs release bioactive factors like VEGF and BDNF. These factors not only promote vascular network formation, remodel the ECM and inhibit tissue fibrosis but also rescue damaged and degenerated neurons at injury sites [85, 86]. Second, another critical mechanism by which BM-MSC to participate in tissue repair is immune regulation [87]. Inflammation initiates the process after skeletal muscle injury. Reducing the duration or intensity of this phase is essential for tissue recovery. Research indicates that BM-MSCs secrete IL-4 and IL-10, which promote the transition of M1 to M2 macrophages, and then, trigger the regression of inflammation [88]. The regenerative potential of BM-MSCs has been shown in the injury tibial anterior muscle. BM-MSCs have been shown to facilitate repair and regeneration four-month post-transplantation [89]. Moreover, BM-MSCs that were subjected to low-oxygen conditions beforehand took root more effectively in damaged muscles; however, they didn't transform into muscle cells [90]. BM-MSCs have the ability to promote the regeneration of damaged muscle. However, the methods to enhance BM-MSCs' survival and differentiation efficiency *in vivo* still require more in-depth research.

### Adipose-derived mesenchymal stem cells

Adipose-derived mesenchymal stem cells (AD-MSCs) are similar to BM-MSCs. They have self-renewal ability and differentiative capacity, are rich in sources and can be easily separated from adipose tissue, and also show promise for use in tissue engineering applications [91]. The collection simplicity and pleiotropy of AD-MSCs make them attractive for adult stem cells to repair damaged tissues and organs [91]. Extracellular vesicles EVs from AD-MSCs can regulate ECM remodeling by upregulating the ratios of collagen III/I, TGF-β3/TGF-β1 and TIMP-1 (MMP-3/MMP-1 tissue inhibitor) [92]. In addition, the myogenic effect of human AD-MSCs indicates that they have the prospect of being used as a source of skeletal muscle regeneration cells [93]. MyoD and myogenin expressed by AD-MSCs are the primary transcription factors that regulate muscle differentiation [10]. AD-MSCs demonstrate the capacity to facilitate myotube formation through close interaction with myoblasts, thereby enhancing the differentiation process and supporting skeletal muscle tissue repair [14]. AD-MSCs can secrete cytokines and growth factors, and show the characteristics of immune regulation and angiogenesis [94]. In addition, studies have shown that the main cytokine secreted by AD-MSCs is IL-6. IL-6 induces M2 macrophage polarization, subsequently suppressing inflammatory responses while stimulating tissue revascularization and muscle regeneration through coordinated molecular mechanisms [95].

### Amniotic fluid stem cells (AFSCs)

AFSCs have a variety of differentiation capacities and nontumorigenic, and can differentiate into six different lineages, including osteogenic, adipogenic, myogenic, neurogenic, endothelial and hepatogenic [10]. AFSCs have high myogenic potential and expresses MyoD and desmin in muscle specific induction medium [14, 96]. The study found that the transplantation of AFSCs into the mouse model of spinal muscular atrophy by tail vein can enhance the muscle strength and improve the survival rate. In addition, only AFSCs transplanted mice could effectively regenerate muscle in tibialis anterior muscle damaged by cardiotoxin. The second transplantation of SCs from the treated mice showed that AFSCs were integrated into the muscle stem cell niche and had no difference with SCs in long-term muscle regeneration [97, 98]. The research shows that AFSC-EVs can inhibit oxidative stress, show immune regulation and neurogenic activity, and protect NMJ and motor neuron during dexamethasone induced muscle atrophy [99]. The potential applications of extensive regenerative medicine and tissue engineering and relatively few ethical issues make AFSCs as promising cells in cell therapy, but so far, the research of AFSCs for muscle regeneration/repair is relatively few.

### Induced pluripotent stem cells (iPSCs)

iPSCs are developed from nonpluripotent cells (usually adult somatic cells) by expression of pluripotent genes, such as Sox-2, Oct-4, c-Myc and Klf4 [98]. The iPSCs show similar transcriptional and epigenetic characteristics to embryonic stem cells (ESCs), which opens up exciting prospects for tissue engineering. The iPSCs have become another attractive source of myogenic cells for muscle regeneration due to the abilities of self-renewal and differentiation. Particularly, through transcriptional activation of core myogenic regulators like Pax7 and MyoD, the iPSCs can undergo direct lineage conversion to generate functional skeletal myocytes [81]. It has been explored to induce iPSCs to differentiate into transplantable MuSCs (iMuSCs) [13]. Studies have shown that transplantation of iMuSCs into the diaphragm can improve the respiratory function of patients with DMD [100]. The iPSCs can also be easily transformed into muscle progenitor cells (MPCs) by using virus vectors or small molecules to overexpress PAX 3/7. When MPCs are implanted *in vivo*, they will fill the niche of stem cells, and then, repair the injured muscle [101]. These cells are easy to expand and exhibit low immunogenicity and negligible tumorigenic potential post-transplantation. The small molecules CHIR99021 and Givinostat were used to successfully generate MPCs from multiple human iPSCs. Givinostat induced MPCs (Givi-MPCs) can improve muscle regeneration in elderly NSG (NOD scid gamma) mice and DMD mice [102, 103]. Moreover, hiPSC-derived myogenic progenitors fabricate highly contractile 3D engineered skeletal muscles with forces similar to primary myoblast-derived tissues through precisely tuned hydrogel and medium conditions [28]. Therefore, iPSCs may be viable alternative to SCs and have great potential in SMTE. However, iPSCs derived from patients are mainly used as *in vitro* models to simulate muscle diseases, study pathological mechanisms and conduct drug testing before *in vivo* [101]. If reliable, efficient and safe muscle generation is achieved, further research is needed.

### C2C12

C2C12 is an immortalized myoblast derived from mouse SCs, which has the characteristics of rapid proliferation and easy transfection. C2C12 is not only an ideal cell model for muscle research but also widely applied in tissue engineering, regenerative medicine and so on. They can rapidly form contractile myotubes and differentiate into skeletal muscle cells, smooth muscle cells and myocardial cells. The most commonly used myoblast cell line in SMTE is the C2C12, because it achieves a fast, reliable and low-cost method [104]. However, there are still many unsolved problems in cell implantation, survival and immune rejection.

### Other cell types

Research has demonstrated the potential of other cell types for muscle tissue engineering applications, including muscle side population (SP) cells, M2 macrophages and hematopoietic stem cells (HSCs). The muscle SP cells have been proved to produce SCs and myoblasts *in vivo* when injected into the injury skeletal muscle [105]. The implantation of M2 macrophages enhances myofiber regeneration through coordinated neovascularization, which demonstrated a viable strategy for functional muscle restoration [106]. In addition, HSCs, perivascular progenitor cells and human umbilical cord blood (UCB)-derived MSCs also show muscle regenerative potential [105, 107, 108]. However, the relevant mechanisms need further research to be better applied to the study of SMTE.

**Table 1.** Seed cells for skeletal muscle tissue engineering

Seed cells	Mechanism of muscle repair	Advantages	Disadvantages	References
SCs	Upon activation, quiescent SCs initiate proliferation to generate MPCs. These MPCs subsequently undergo differentiation into myoblasts, which fuse to form multinucleated myotubes. Through maturation processes, these syncytial structures ultimately develop into muscle fibers.	A natural precursor of muscle tissue	High cost; low separation rate; complex purification operation; limited proliferation capacity *in vitro*	[81]
BM-MSCs	BDNF, IGF-1, VEGF and S1P were secreted by BM-MSCs to promote the proliferation and differentiation of SCs; VEGF derived from BM-MSCs has been proved to upregulate mTOR expression through PI3K/AKT or JAK/STAT3 signaling pathway and promote the differentiation of myoblasts.	Easy to obtain; *in vitro* good expansion capacity; good immune regulation	Low *in vivo* survival rate and differentiation rate	[84–86, 88]
AD-MSCs	MyoD and myogenin expressed by AD-MSCs are the main transcription factors that regulate skeletal muscle differentiation; The direct contact between AD-MSCs and myoblasts can induce them to fuse into myotubes, and promote cell differentiation and skeletal muscle regeneration.	Good Self-renewal and versatility; rich in resources	The myogenic capacity was weaker than that of skeletal muscle-derived cells	[10, 94, 95]
AFSCs	AFSCs has high myogenic potential and expresses MyoD and desmin in muscle specific induction medium.	General versatility and nontumorigenicity; few ethical issues	Further research of myogenic induction protocol is needed	[14, 99]
iPSCs	Particularly through transcriptional activation of core myogenic regulators like Pax7 and MyoD, the iPSCs can undergo direct lineage conversion to generate functional skeletal myocytes.	The capacity for unlimited self-renewal; the ability to differentiate into myogenic cells	Complex differentiation process; the residual undifferentiated cells have a high risk of tumorigenesis	[101–103]
C2C12	C2C12 can rapidly form contractile myotubes and differentiate into skeletal muscle cells, smooth muscle cells and myocardial cells.	Pluripotency; rapid proliferation; easy transfection; low cost	Non-human, xenograft immune rejection	[104]
Muscle SP cells	Muscle SP cells have been proved to produce SCs and myoblasts *in vivo* when injected into the injury skeletal muscle.	The potential of muscle regeneration	Difficult isolation and purification; *in vivo* homing mechanism is unknown	[105]

## Biomaterials for tissue engineering of skeletal muscle

Biomaterials hold broad prospects for repairing and restoring damaged muscle tissue in skeletal muscle regeneration. The basic function of biomaterials is to mimic the natural environment, provide chemical and physical cues for transplanted or host muscle cells, promote their functional maturity, prevent body reactions and create a local niche that can develop muscle successfully [19]. Biomaterials serve as scaffolds for muscle tissue growth in the damaged area. These scaffolds can provide a synthetic extracellular matrix for various cells. Among them, 3D muscle tissue has been designed to more closely imitate natural tissue and plays a vital role in skeletal muscle regeneration [93]. The composition of biomaterials and their biomechanical effects and functions are important. Biomaterials can be designed to deliver a variety of biological factors, such as growth factors, cells, miRNAs, drugs and other molecules, directly to the injury site, enhancing the regeneration ability, promoting healing and facilitating the formation of new muscle tissues [23, 109]. The biomaterials should show biocompatibility, helping transplanted tissue integrate into host tissue, supporting blood vessel formation, enhancing nerve development. They can also be designed to replicate the structure and mechanical properties of natural muscle tissue, guiding the growth and organization of new muscle fibers [110]. In the case of large-scale muscle defect, biomaterials can fill in the defect and stop fibrotic tissue from forming, so as to prevent the functional damage caused by the loss of muscle tissue.

Hydrogel is a kind of hydrophilic polymer with high water content, microporous structure, biocompatibility and adjustable mechanical properties. It is an excellent candidate for drug and cell delivery carriers, and provides a bionic microenvironment suitable for skeletal muscle, due to its mild processing conditions, similarity to natural tissue extracellular matrix and less invasive delivery. It is the preferred biomaterial for skeletal muscle tissue regeneration [111–113]. Encapsulation of muscle cells in hydrogel can promote differentiation and regeneration of skeletal muscle tissue. In addition, embedding bioactive molecules (IGF-1, bFGF-2, VEGF) into the hydrogel matrix and directly implanting into the damaged site can create a biochemical microenvironment conducive to supporting the functional regeneration of skeletal muscle, and also provide a positive impact on muscle precursors by improving proliferation, adhesion and differentiation [114]. Hydrogel can also combine with specific growth factors, promote the repair of blood vessels and nerve tissues, helping to accelerate the processes of regeneration and integration of skeletal muscle tissue [115, 116]. The hydrogels have great potential in tissue regeneration of skeletal muscle, it will be a fascinating endeavor for researchers to develop novel and innovative biopolymer hydrogels with better performance. Biomaterials for SMTE are mainly divided into three categories: natural, synthetic and composite materials (Table 2).

### Natural materials

**Figure 2.**
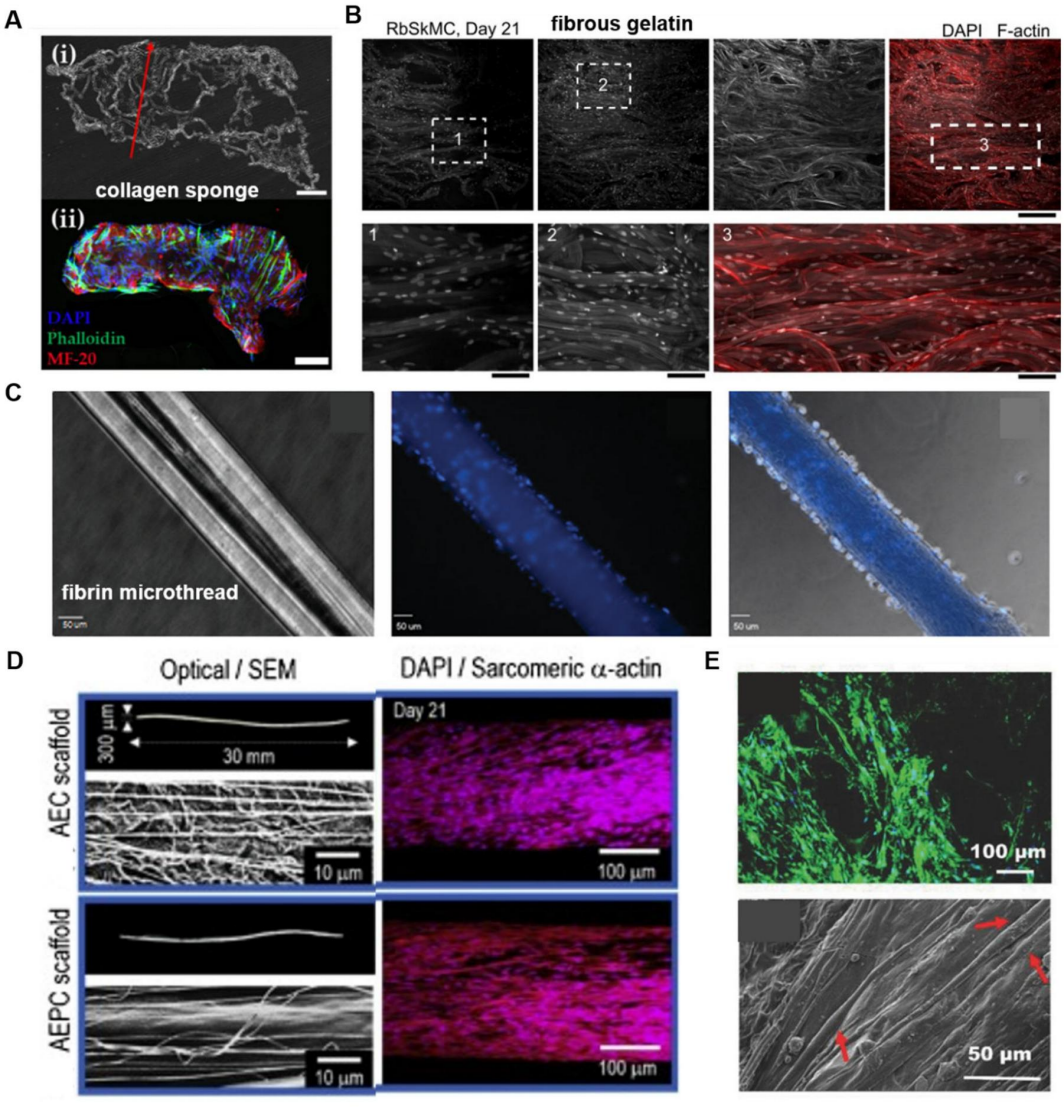
Natural materials for skeletal muscle tissue engineering. (**A**) Representative histological section of a collagen sponge, (**i**) brightfield image showing the angle of pore alignment (red arrow), (**ii**) immunostaining demonstrating myoblast alignment within a longitudinal section of collagen sponge. Scale: 500 µm [120]. Copyright 2023, MDPI. (**B**) Rabbit skeletal muscle myoblast cells (RbSkMC) cultured in fibrous gelatin. Immunofluorescent staining of cell nuclei (DAPI, white) and cytoskeletal actin filaments (F-actin, red). Gelatin fibers appear light gray in the DAPI channel. Magnified views reveal cell nuclei anisotropy and alignment with the underlying gelatin fibers; scale bars are 200 μm (top row) and 50 μm (bottom row) [124]. Copyright 2019, Springer. (**C**) The fibrin microthread before cell seeding (left), after seeding with muscle-derived fibroblasts (Hoechst 33342-blue, middle) and phase-contrast merged image (right). Scale bar: 50mm [126]. Copyright 2011, Mary Ann Liebert. (**D**) The optical and SEM images of the alginate-electrospun collagen (AEC) and alginate-electrospun PVA-leached collagen (AEPC) scaffolds (left). The fluorescence images (nuclei/sarcomeric α-actin) indicate that the myoblasts were well aligned in the topographical direction after 21 d of culturing, and the myotubes were well developed (right) [130]. Copyright 2019, Elsevier. (**E**) C2C12 differentiation on SF/PASA2 (2% PASA, w/w) scaffolds. The fluorescent merged image (MHC-green, above) and SEM image (below) of SF/PASA2. Cells are indicated by arrows [135]. Copyright 2017, John Wiley and Sons.

Natural materials have inherent biocompatibility and degradability, which contribute to cell attachment, migration, interaction, cell proliferation and differentiation [117]. However, natural materials have the disadvantages of poor mechanical properties and no ordered structure [93]. Natural materials for SMTE include decellularized ECM from various organs and donors, ECM components and other natural derived materials, such as collagen, gelatin, fibrin, alginate, hyaluronic acid, silk fibroin, agarose, chitosan and laminin (Figure 2).

Collagen, the major component of ECM in many tissues, has good biodegradability and biocompatibility, can support cell adhesion, promote cell proliferation and tissue healing, and induce cell polarization [118, 119]. Due to these characteristics, collagen is widely used in tissue engineering. Research indicated that collagen scaffolds facilitate myoblast infiltration and differentiation into muscle fibers, as well as the revascularization of injured muscle tissues [120, 121]. In addition, gelatin is a kind of fibrous protein produced by denaturation and partial hydrolysis of collagen, which is a denatured form of collagen. Gelatin is a biocompatible material, which not only has natural cell binding motif (RGD) and matrix metalloproteinase (MMP) sequences, providing cell binding sites and biodegradability but also has good tissue regeneration characteristics and superior biomechanical properties, and is widely applied as scaffold material in tissue engineering [122–124]. In the current research, it is often esterified by methacrylate to form a photosensitive methacrylated gelatin (GelMA), which can enhance the structural stability and mechanical stability of the scaffold [123]. Fibrin is another natural material for SMTE, forming a hydrogel network by the polymerization of fibrinogen, which shows rich cell binding and degradation motifs and the ability to bind cell-derived growth factors [123]. In addition, fibrin has the specific binding site for angiogenic factors. In addition to stimulating angiogenesis, fibrin can also act as temporary wound healing matrix, naturally participate in wound healing and tissue repair, and improve myogenesis [113]. Myoblasts can evenly diffuse in the fibrin scaffold, reshape the network and differentiate into mature muscle fibers [123]. More than 10 years ago, it was reported that primary myoblasts were transported to skeletal muscle defects of rat using fibrin hydrogel, and the injected myoblasts increased their integration with host muscle fibers in a local and time-dependent manner [125]. Subsequent studies also found that fibrin microfilament scaffolds supported the growth of myofibrils and improved the regeneration of large-scale defects of skeletal muscle in mice of VML model [126, 127]. Moreover, the combination of PEGylated fibrin with a lipid nanoparticle might speed up the translation of injectable hydrogel-based RNA interference in skeletal muscle [128]. Alginate is a biocompatible and biodegradable polysaccharide derived from brown algae, and it has low inflammatory immune response. However, alginate lacks RGD binding site and MMP biodegradable sequence, which limits the cell activity in the scaffold [123, 129]. In the layered structure obtained by alginate nanofibers and polycaprolactone (PCL) pillars, alginate nanofibers can promote the arrangement of myoblasts [130]. The multi-hierarchical tissue-like structure constructed by aligned shear-patterned sodium alginate microfibers and cells along the fiber was fabricated based on wet-spinning method. The potentially novel constructing provides a new idea for the engineered linear muscle tissue [131]. Hyaluronic acid (HA) is the key component of ECM, with excellent biocompatibility and anti-inflammatory properties [132]. Studies have shown that HA serves as a critical modulator in muscular regeneration processes, primarily through stimulating myoblast migration and differentiation pathways and facilitating the mobilization of muscle progenitor cells [133]. In addition, silk fibroin (SF) is a naturally occurring protein in scaffold materials. Owing to its excellent mechanical properties, biocompatibility, biodegradability and bioabsorbability [134]. The electroconductive scaffolds based on SF and water-soluble conductive poly(aniline-co-N-(4-sulfophenyl) aniline) (PASA) demonstrate myogenic potentiation efficacy in C2C12 lineages, presenting as a tunable bioerodible platform for volumetric muscle defect restitution [135]. SF is usually dissolved in aqueous solution in applications of tissue engineering, which can be made as films, hydrogels and sponges by various manufacturing technologies.

In addition, many types of hydrogels are used in the research of skeletal muscle repair and regeneration. Common natural hydrogels, such as collagen hydrogel, fibrin hydrogel, gelatin hydrogel and alginate hydrogel, possess biocompatibility, biodegradability, low inflammation and bioactive sites for cell adhesion and biodegradation, and often play vital roles in muscle regeneration. However, they cannot perfectly imitate the biochemical, structural and mechanical complexity of ECM [117]. Decellularized ECM hydrogel promotes skeletal muscle injury repair. The porcine dECM hydrogel demonstrates multifaceted regenerative capacity in myofibral reconstruction, immunomodulation and microenvironment remodeling through normalizing iron metabolism and effectively restoring macrophage immune homeostasis [136]. The oxidized alginate-gelatin hydrogel, as a simple, cost-effective and biodegradable biological ink, has been successfully used in C2C12 culture *in vitro* in muscle engineering [137]. ROS scavenging hydrogel containing MSCs can promote the regeneration and restore the motor function of damaged skeletal muscle by promoting MSCs expansion, inhibiting inflammation and myogenic differentiation [138]. The C2C12 myoblasts organize and fuse into aligned myotubes with spans of several millimeters, which cultured on a highly compliant isotropic gelatin hydrogel [139]. In rat model of anterior tibial VML injury, the robust functional recovery observed in anterior tibial after implantation of semisynthetic HA-based hydrogel at the injured site, accompanied by volume reconstruction, muscle regeneration and quasi-natural vascularization [140]. The photopolymerized HA hydrogels also have good prospects in the case of VML injury, due to their fast gel kinetics, easy delivery, compliance with wound sites and high level of chemical adjustability. The HA hydrogels with intermediate stiffness were found to promote myogenesis while reducing the chronic inflammatory response characteristic of VML injuries [141].

At present, natural materials from mammals (such as collagen, fibrin and gelatin) are more appropriate for SMTE, because they have higher cell adhesion density, higher cell proliferation rate, hydrogel compaction, endogenous growth factors and biological signals for differentiation [142]. Moreover, extracellular vesicles (EVs), acting as key mediators of intercellular communication, received much attention in tissue engineering, but have not been reported in muscle tissue engineering. The protective and repairing effects of EVs on muscles have been positively reported [4]. The more studies are expected to add EVs to biomaterials to directly or indirectly promote muscle regeneration. 

### Synthetic materials

**Figure 3.**
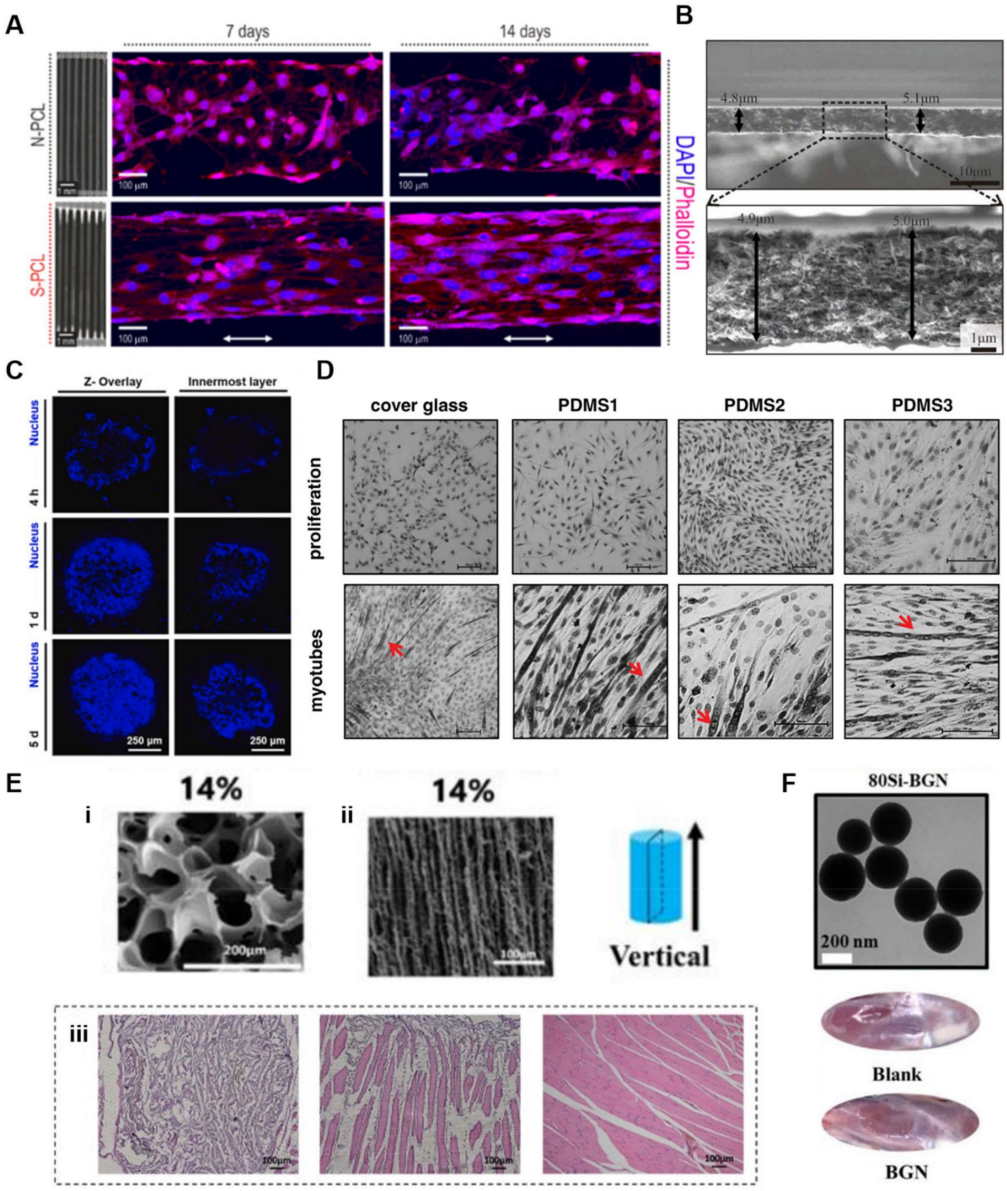
Synthetic materials for skeletal muscle tissue engineering. (**A**) Fluorescence images of myoblasts after 7 and 14 d stained with DAPI (blue) and phalloidin (red). Randomly oriented cells were observed on nonstretched PCL (N-PCL), whereas the uniaxially aligned myoblasts were present on stretched PCL (S-PCL) [146]. Copyright 2019, IOP Publishing. (**B**) SEM images at the cross section of PEG-CNT films, top: 2000× magnification, bottom: zoom-in view at 10 000× magnification [144]. Copyright 2015, RSC Pub. (**C**) A confocal laser scanning microscope (CLSM) images illustrating adhesion and initial proliferation of C2C12 cells cultured dynamically in the PLGA porous microspheres (PMs) [151]. Copyright 2021, Elsevier. (**D**) Fusion of C2C12 cells and primary myoblasts cultured on PDMS substrates with varying elasticity (PDMS1: Shore hardness 55, PDMS2: Shore hardness 40, PDMS3: Shore hardness 70). C2C12 cells stained with Pappenheim's method. Red arrows indicate multinucleated myotubes. Scale bar: 100 μm [145]. Copyright 2019, John Wiley and Sons. (**E**) (**i**) SEM photographs of random porous PU (14 wt %) scaffolds; (**ii**) SEM photographs of aligned porous PU (14 wt %) scaffolds; (**iii**): H&E staining of regenerated muscle tissue 2 weeks post-implantation. Left: random porous PU scaffold, middle: aligned porous PU scaffold, right: healthy muscle soft tissue [152]. Copyright 2019, Oxford University Press. (**F**) The TEM images of 80Si-BGN (top), and histological observation of skeletal muscle tissue repair by 80Si-BGN after 4 weeks (bottom) [153]. Copyright 2023, Oxford University Press.

The synthetic materials possess tensile mechanical properties and structural characteristics that are repeatable and easy to adjust, and can imitate the natural mechanical properties of skeletal muscle as well as create chemical and physical environments conducive to cell growth. However, the synthetic materials lack the functional sequences recognized by cells, which hinder the attachment and growth of cells on surfaces [14, 132]. At present, many synthetic materials have been used in tissue engineering of bone, skin, muscle, nerve, blood vessel, tendon, liver and kidney [143]. However, there are not many synthetic polymers used in the research of SMTE, including poly-ε-caprolactone (PCL), polyethylene glycol (PEG), poly (lactic-co-glycolic acid) (PLGA) and polydimethylsiloxane (PDMS) (Figure 3) [74, 130, 144, 145]. Among them, PCL, PEG and PLGA are commonly used biodegradable and highly biocompatible materials in biomedical applications approved by FDA.

PCL as a pillar can enhance the mechanical strength of the scaffold [130]. The scaffold made from PCL with uniaxially aligned surface topography has been shown to increase the proliferation and differentiation of myoblasts cultured on it [146]. Moreover, PCL multicompartmental scaffolds were created with aligned fibers via electrospinning and mechanical stretching, and subsequently surface-functionalized with the cell-supporting tropoelastin, attached using physisorption or covalent attachment. The scaffolds can be fabricated to imitate tendon organization [147]. PEG can serve as scaffold of hydrophilicity, and its stiffness can be easily changed according to the chain length, concentration and quantity [148]. The PEG-linked multiwalled carbon nanotube (PEG-CNT) films demonstrated myogenic lineage-specific commitment in hMSCs and can work as a promising material towards skeletal muscle injury repair [144]. In addition, the research showed that methacrylic acid (MAA)-PEG hydrogel with the slow and rapid degradation not only increased the expressions of TNF-α and IL-10 but also increased the levels of M2 macrophage markers. The MAA hydrogel reduced the number of pro-inflammatory MHC II^+^ macrophages in skeletal muscle. However, whether the slowly degraded MAA hydrogel can contribute to skeletal muscle regeneration needs further research [149]. Studies have shown that ADSCs attached to PLGA spheres will generate muscle tissue when subcutaneously injected into nude mice [150]. An injectable PLGA porous microspheres (PLGA PMs) had facilitated the cell adhesion and proliferation of C2C12 cells. In a mouse VML model, consisting of C2C12-laden PLGA PMs and human umbilical vein endothelial cell-laden PEG hollow microrods (PEG HMs) improved *in situ* muscle regeneration and remolding [151].

Furthermore, PDMS is highly hydrophobic, has good optical, electrical, mechanical properties and biocompatibility, and can promote myoblast differentiation and myotube formation [145]. Polyurethanes (PUs) are common biodegradable materials applied in medical implant. The aligned 3D porous polyurethane (PU) scaffold obtained from directional freeze–drying method preserves its structural alignment and demonstrates capacity to guide the ordered repair and regeneration of muscle tissue, even it was partly degraded *in vivo* for 2 weeks [152]. Bioactive glass nanoparticles (BGNs) are extensively employed in biomedical applications. However, limited data exist regarding their biological influences on SMTE. The BGNs demonstrated the ability to effectively enhance myogenic differentiation in C2C12 cells. *In vivo* studies using a rat skeletal muscle defect model further validated that 80Si-BGN significantly enhanced complete skeletal muscle regeneration during 4 weeks implantation [153]. Two-dimensional Ti_3_C_2_T_X_ (MXene) exhibits unique photoelectromagnetic characteristics and facilitates angiogenesis, muscle fiber formation and skeletal muscle regeneration via modulation of the cellular microenvironment through anti-inflammatory and antioxidant mechanisms in rats with tibialis anterior muscle defects [154]. In addition, synthetic hydrogels, such as PEG and polyacrylamide (PAAm), have excellent mechanical properties and can better control scaffold structure and chemical composition. However, due to their lack of biological molecules, they need to be modified to support adhesion, differentiation and vitality of cells [148]. Therefore, compared with natural hydrogel, synthetic hydrogels need to be modified for tissue regeneration in skeletal muscle.

### Composite materials

**Figure 4.**
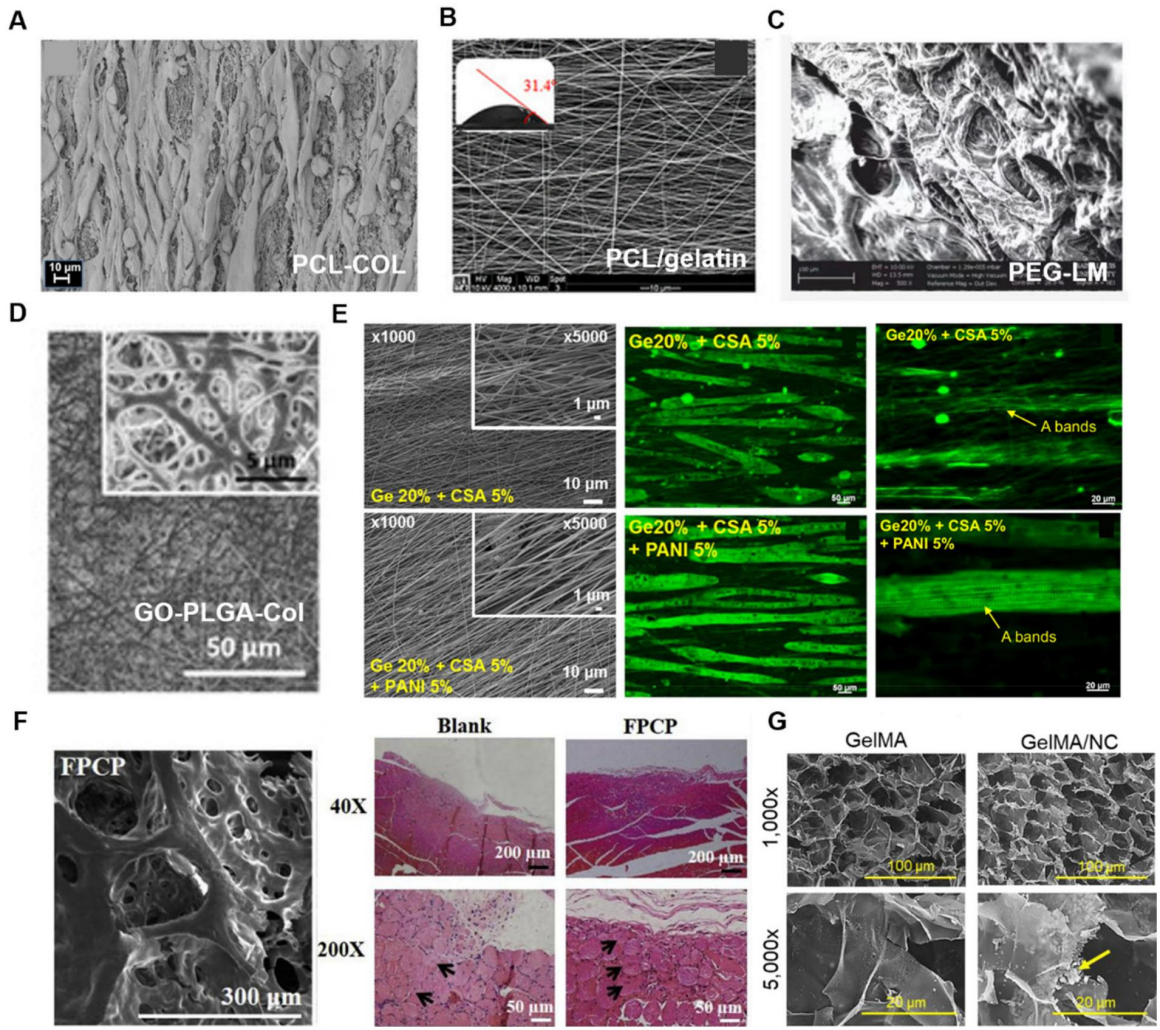
Composite materials for skeletal muscle tissue engineering. (**A**) SEM of C2C12 on PCL-collagen I nanoscaffolds after 28 days of myogenic differentiation showed parallel alignment with myotube-like structures [155]. Copyright 2018, Springer. (**B**) Contact Angle and SEM images of the PCL/gelatin scaffolds with varying fiber alignments [156]. Copyright 2021, John Wiley and Sons. (**C**) SEM of Laminin-111 functionalized PEG hydrogels [148]. Copyright 2018, IOP Publishing. (**D**) FE-SEM images of GO-PLGA-Col fiber matrices [157]. Copyright 2015, Springer. (**E**) FE-SEM images of the gelatin 20% + CSA 5% and gelatin 20% + CSA 5% + PANI 5% nanofibers electrospun obtained at 50°C, and 18 kV (left); Myotube formation at day 6 in differentiation medium was observed via fluorescence microscopy (fast skeletal myosin, green) on aligned electrospun (middle); “A bands” formation in myotubes at day 4 in differentiation medium was also visualized using fluorescence microscopy (fast skeletal myosin, green) on aligned electrospun (right) [164]. Copyright 2017, American Chemical Society. (**F**) TEM images of FPCP hydrogel (left); Skeletal muscle regeneration *in vivo* after treated with FPCP hydrogel for 4 weeks. H&E staining pictures of skeletal muscle defect area reveals centronucleated myofibers (black arrows, right) [165]. Copyright 2021, Elsevier. (**G**) SEM micrographs of GelMA and GelMA/NC demonstrated similar porous morphologies at 1000× and 5000× (scale bars of 100 μm and 20 μm, respectively). Pore sizes were of the same order of magnitude. The GelMA/NC group did display powdered clusters of NC (yellow arrow) in between GelMA layers as demonstrated at 5000× [168]. Copyright 2022, RSC Pub.

Due to the low biological activity of these synthetic materials, most recent studies have combined natural and synthetic polymers to form composite materials to produce nanofibers with appropriate physical, chemical and biological properties, so that the composite materials possess the mechanical, conductive and biocompatible properties required for the regeneration of skeletal muscle tissue (Figure 4). For example, the PCL-collagen nano-scaffold can facilitate the myogenic differentiation of MSCs and myoblasts in serum-free medium [155]. In addition, the mixed nanofibrin-PCL scaffolds containing varying gelatin/collagen concentrations were fabricated via electrospinning, demonstrating potential applicability in SMTE [156]. The hydrogel composed of PEG and laminin-111 can promote the adhesion and survival of myoblasts, the production of growth factors and myogenic activity, and provide the elastic matrix for the proliferation of SCs at the injury site [148]. Furthermore, hybrid fiber matrices composed of PLGA and collagen (Col) impregnated with GO (GO-PLGA-Col) stimulate myoblasts differentiation for skeletal muscle regeneration and exhibit superior bioactivity and biocompatibility [157]. Nanofiber hydrogel composites have also been shown to promote the polarization of M2 macrophages, create a favorable immune microenvironment and enhance the repair of VML damage [158]. Moreover, the SrCO_3_/CaSiO_3_/alginate composite hydrogel can not only promote capillary regeneration but also directly promote the expression of MyoG and MyoD, enhance M2 polarization of macrophages, reduce inflammation, promote vascularization and protect skeletal muscle. This hydrogel has the potential to repair defective muscle tissue [159].

In addition to combining natural materials with synthetic materials, these biomaterials may further be integrated with other polymers to construct composite materials, such as conductive polymers, nanofibers or photopolymers, so as to obtain the mechanical, conductive and biocompatible properties required for the regeneration of skeletal muscle [160]. Because skeletal muscle cells respond to electrical stimulation, and electrical signals are vital for cell communication, cell proliferation, cell differentiation and tissue maturation [161]. The conductive biomaterials for SMTE have recently been developed, such as polyaniline (PANI), polypyrrole (PPy), metal nanomaterials and carbon-based nanomaterials (mainly graphene and carbon nanotubes). These conductive biomaterials show great potential in helping repair of skeletal muscle tissue due to their good biocompatibility and promoting cell adhesion, cell proliferation and myogenic differentiation [73, 135, 162, 163]. Composite hydrogel is regarded as the optimal candidate for skeletal muscle regeneration.

The conductive hydrogel developed by combining with conductive polymers such as PANI has been proved to be conducive to regulating the adhesion, diffusion and differentiation of muscle precursor cells, and becomes a suitable option for 3D biomaterials in SMTE [114]. It is reported that compared to gelatin nanofibers doped with camphorsulfonic acid (CSA), enhanced myotube formation and enhanced myotube maturation on composite gelatin-CSA-PANI nanofibers were observed [164]. Moreover, the multifunctional nanomatrix composed of PPy@polydopamine (PPy@PDA, 342 ± 5.6 nm) nanoparticles-crosslinked Pluronic F-127 (F127)-polycitrate matrix (FPCP) could significantly enhance the proliferation and myogenic differentiation of C2C12 cells. Based on the multifunctional properties, FPCP nanomatrix further accelerated complete skeletal muscle repair and regeneration *in vivo* by activating angiogenesis and stimulating myotube formation [165]. The hydrogel composed of GelMA and poly (3,4-ethylenedioxythiophene) (PEDOT) nanoparticles encapsulating C2C12 cells demonstrated efficacy in promoting proliferation and differentiation of C2C12 cells [166]. In addition, GelMA hydrogel, as photopolymer that can facilitate the differentiation of skeletal muscle cells. The research developed and prepared an interpenetrating network (IPN) hydrogel with rapid stress relaxation as biological ink combined with GelMA and fibrinogen through photo-crosslinking and enzyme-crosslinking. C2C12 myoblasts were encapsulated in the IPN hydrogels, and IPN hydrogels showed higher 3D cell proliferation and differentiation [167]. The nanoengineered scaffold based on functionalized GelMA hydrogel was designed to enhance muscle progenitor proliferation and differentiation. The scaffold was capable of controlled release of IGF-1, an important myogenic growth factor, by utilizing the electrostatic interactions with LAPONITE^®^ nanoclays (NCs), providing a potential therapeutic strategy for severe muscle injuries [168]. All these materials have their own advantages and disadvantages. How to use their characteristics to better apply to the SMTE remains a major challenge.

**Table 2.** Biomaterials for skeletal muscle tissue engineering

Biomaterials		Features	Function	Degradation property	References
Natural materials	Collagen	Good biocompatibility; supports cell attachment; promotes cell proliferation and tissue healing	Support myoblasts to infiltrate and differentiate into muscle fibers; revascularization	Good biodegradability	[118, 120, 169, 170]
	Gelatin	Provide cell binding sites; good tissue regeneration properties; excellent biomechanical properties	Support C2C12 myoblasts to fuse into aligned myotubes	Good biodegradability	[122, 123, 139, 156]
	Fibrin	The ability of cells to bind and degrade motifs; the ability to bind cell-derived growth factors	Stimulates angiogenesis; acts as a temporary wound-healing matrix; assists myoblast differentiation; support the growth of myofibrils	Good biodegradability	[113, 123, 126, 127]
	Alginate	Biocompatibility; lower inflammatory immune response	Promote the alignment of myoblasts	Good biodegradability	[123, 129, 130]
	Hyaluronan	Biocompatibility; anti-inflammatory	Upregulation of myoblast migration, differentiation; enhanced recruitment of muscle progenitor cells; volume reconstruction; muscle regeneration	Good biodegradability	[132, 133, 140]
	Silk fibroin	Excellent mechanical properties; biocompatibility; bioabsorbability	working in many forms (films, hydrogels, sponges)	Slow biodegradability	[134]
	Oxidized alginate-gelatin hydrogel	Simple and cost-effective	C2C12 cell culture *in vitro*	Good biodegradability	[137]
Synthetic materials	PCL	Highly biocompatibility	As a support: to enhance the mechanical strength of the stent	Slow biodegradability	[130, 148]
	PLGA	Highly biocompatibility	Helps the ADSC produce muscle tissue	Good biodegradability	[150]
	PEG	Highly biocompatibility; hydrophilicity	Change the stiffness of the bracket	Good biodegradability	[130, 148]
	PDMS	Highly hydrophobic; biocompatibility; good optical, electrical and mechanical properties;	Promote myoblast differentiation and myotube formation	Non-biodegradable	[145]
Composite materials	PCL-collagen nanoscaffolds	Good mechanical properties; biocompatibility	Promote the myogenic differentiation of mesenchymal stromal cells and myoblasts	Slow biodegradability	[155]
	PEG-Laminin-111 hydrogel	Highly biocompatibility; hydrophilicity; provide active sites for cell adhesion	Promote myoblast adhesion and survival; produce regeneration-promoting growth factor; provide elastic matrix for satellite cell proliferation at the injured site	Good biodegradability	[148]
	Methacrylic acid PEG (MAA-PEG) hydrogel	Highly; biocompatibility; hydrophilicity; mechanical properties	Increased M2 macrophages; anti-inflammation	Good biodegradability	[149]
	SrCO3/Casio3/alginate composite hydrogel	Biocompatibility; lower inflammatory immune response; Injectable	Promote capillary regeneration; promote the expression of MyoG and MyoD; reduce inflammation	Good biodegradability	[159]
	GelMA hydrogel	Provide cell binding sites; good tissue regeneration properties; excellent biomechanical properties; structural and mechanical stability	A compressive load supporting skeletal muscle; provide a cell-binding site	Good biodegradability	[168]
	Nanofiber hydrogel	High porosity; biocompatibility	Promote M2 phenotype polarization of macrophages; create favorable immune microenvironment	Good biodegradability	[158]
	GelMA-PEDOT NP hydrogel	High biocompatibility; adjustable mechanical properties; electrical conductivity	Proliferation of muscle fibers	Good biodegradability	[171]
	IPN hydrogel	Fast stress relaxation	Higher 3D cell proliferation and differentiation	Good biodegradability	[167]

## Advanced technology of skeletal muscle tissue engineering

### Conditions for scaffold design

In addition to the characteristics of biocompatibility, biodegradability and mechanical properties, the scaffold for SMTE also requires high elasticity and extensibility, because it is responsible for the generation of movement and strength in the body [73]. First, the scaffold needs to serve as a structural framework for muscle differentiation and generation, to help form orderly, dense and well-oriented muscle fibers [113]. Then, the biocompatibility of the scaffold requires that it must support appropriate cell activities, without cytotoxicity and inflammatory reaction. The biodegradability requires that the scaffold will be gradually replaced by newly formed muscle and avoids the occurrence of infection related to the long-term presence in the body [132, 172]. Second, mechanical properties play a fundamental role in SMTE. Among them, matrix stiffness can conduct mechanical transduction and activate intracellular signal transduction, thus, affecting the key functions of muscle precursor cells [114]. The stiffness of skeletal muscle ranging between 12 and 16 kPa, and skeletal muscle deforms during contraction. Therefore, it is necessary to select the appropriate stiffness of the scaffold to make it have the deformation ability similar to that of skeletal muscle, and better facilitating the formation of new muscle fibers that can be integrated into healthy tissues [173, 174]. Porosity is essential not only for the removal of waste and the full diffusion of nutrients, regulatory factors in cells and gases but also for cell encapsulation, myoblast infiltration and inward growth of muscle fibers [112, 172, 175]. Research shows that multiscale porosity also provides multiscale bionic mechanical properties to achieve better tissue integration, muscle formation and functional muscle recovery [176]. Finally, the conductivity of the scaffold is also important. Appropriate artificial electrical stimulation can reorganize the cytoskeleton, induce the directional migration of cells in the direction of the electric field, and promote the transformation of myogenic cells to functional muscle fibers [114, 173]. Furthermore, muscle enhancement and support therapy devices have contributed significantly to the advancement of muscle tissue engineering [177]. With the aim of better promoting the development of skeletal muscle, the optimal manufacturing conditions and parameters should be properly selected for each characteristic.

### Synthesis technology for scaffold

**Figure 5.**
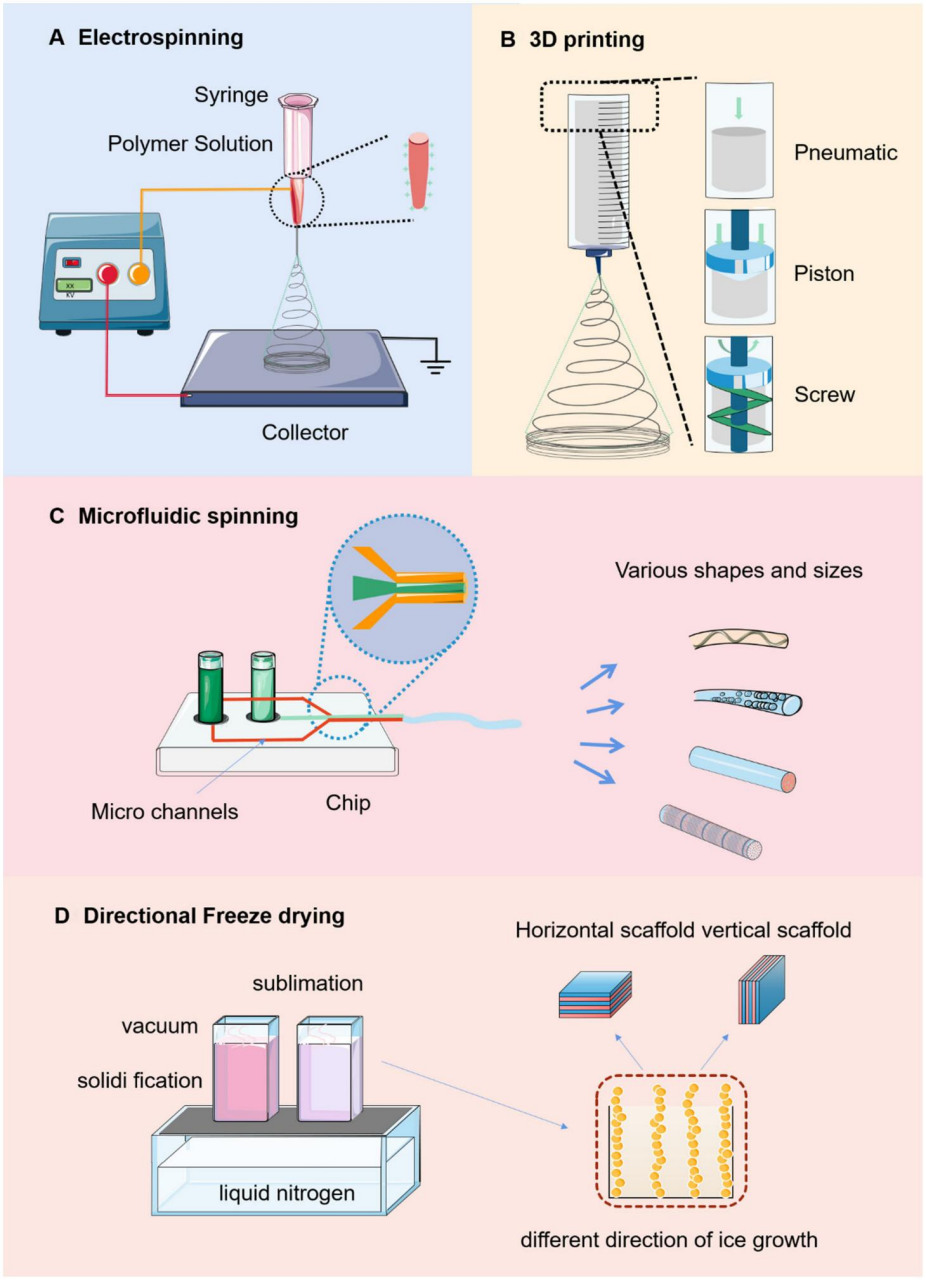
Advanced technology of skeletal muscle tissue engineering. (**A**) Electrospinning: A high voltage is applied to a polymer solution contained in a syringe, which is then extruded through a nozzle onto a collector. The electric field causes the polymer jet to stretch and solidify into fibers as it travels towards the collector. (**B**) Three-dimensional printing: The printer uses a pneumatic system to control the flow of the bioink through a nozzle, which is moved by a screw mechanism to precisely position the material. The piston applies pressure to the bioink, allowing it to be extruded and deposited in the desired pattern. (**C**) Microfluidic spinning technology: It utilizes microchannels to control the flow of polymer solutions and create fibers with precise control over their shape and size. (**D**) Freeze-drying: The scaffold is placed in a vacuum chamber filled with liquid nitrogen, and the ice crystals within the scaffold grow in a specific direction due to the temperature gradient to generate a scaffold with a highly interconnected pore structure.

The technologies used for the synthesis of scaffolds in SMTE include electrospinning, 3D bioprinting, freeze-drying and microfluidic spinning [124, 178, 179] (Figure 5). Among them, electrospinning and 3D printing are common synthesis technologies used in scaffold. Electrospinning is widely used to manufacture nanofiber, which can be used to manufacture uniform scaffolds with required characteristics, including mechanical properties, porosity, orientation and structure similar to ECM in natural tissues. To achieve the best mechanical and physicochemical properties, the combination of synthetic and natural polymers is usually required [156, 180]. Electrospinning is widely utilized in SMTE due to its ability to produce anisotropic and geometrically aligned nanofibers, which effectively guides the formation of aligned muscle fibers [11, 181] (Figure 5A). The scaffolds of PCL/elastin fabricated via electrospinning has been employed as supports for muscle cells [182]. In addition, early studies have shown that electrospun fiber scaffolds arranged with conductive polymers may stimulate muscle cell differentiation due to their fiber arrangement and electrical conductivity, and have a synergistic effect on myogenesis of myoblasts [183, 184]. PCL/PANi nanofiber scaffold fabricated by electrospinning has become a suitable scaffold material for bone tissue engineering [184]. In addition, carbon nanotubes are structural materials with high conductivity, high surface area, good flexibility and resilience. Electrospinning was found to be conducive to the arrangement of carbon nanotubes in the fibers. When carbon nanotubes were incorporated into the polymer matrix, they showed good compatibility with myoblasts, which made them a new scaffold for SMTE [185, 186]. Studies have shown that scaffolds of single-walled carbon nanotubes (SWNTs) or multiwalled carbon nanotubes (MWNTs) combined with polyurethane by electrospinning facilitate the formation of multinucleated C2C12 myotubes more effectively than nonconductive scaffolds [187].

Three-dimensional printing is an effective tool for reconstructing skeletal muscle tissue. It can provide sufficient myogenic microenvironment and required directivity for muscle, and well simulate the directional bundle structure of natural skeletal muscle [188–191]. A primary obstacle confronting 3D printing of skeletal muscle is the limited biomaterials with elastic, durable and biodegradable characteristics, which requires the synthesis of new copolymers to apply to the tissue properties [192]. Natural or synthetic polymers, hydrogels (alginate, collagen, gelatin), decellularized ECM and their composites have been extensively applied as bioinks for 3D bioprinting of engineered skeletal muscle tissues [58, 193, 194]. Hydrogel provides an appropriate microenvironment for differentiation and maturation of cells during the process of 3D printing, while the anisotropic structure of 3D bioprinting provides 3D macroenvironment for myotube tissue [167]. The gelatin hydrogel matrix with macro-patterns produced by extrusion 3D printing is adequate to guide large-scale self-assembly of myoblasts into aligned and extended myotubes [195] (Figure 5B). Xanthan gum and fibrinogen hydrogel (CELLINK^®^ GelXA FIBRIN) and nanofibrillated cellulose (NFC)/alginate fibrinogen hydrogel (CELLINK^®^ FIBRIN) are commercially available hydrogels of bioink. After 21–28 days of culture in the hydrogel, muscle derived cells can fuse together to form structurally aligned myotubes, and show high expression levels of specific skeletal muscle markers, such as MyoD1 and MCK, showing the better potential to support the long-term differentiation of muscle cells in 3D structure *in vitro* [196]. On the basis of IPN hydrogel, the new research further prepared 3D aligned biomimetic scaffolds by using 3D printing gel strategy, successfully cultured skeletal muscle tissue *in vitro*, and was able to recruit native muscle cells and promote *in situ* revascularization to enhance repair of VML model *in vivo* [167]. Moreover, a study also expanded upon a new approach of multimodal bioprinting which enabled the fabrication of thick hierarchical vascular muscle flaps composed of bioprinted and vascularized skeletal muscle tissue, and a 3D-printed engineered macrovessel, which successfully repaired VML injury *in vivo* [197]. This opens up a new way for the research on skeletal muscle biomimetic scaffold. The muscle decellularized ECM (mdECM) bio-ink and 3D cell printing technology will provide appropriate muscle tissue structure [198]. Three-dimensional printing technology has become a powerful tool for construction of engineering skeletal muscle, but its shortcomings such as high cost, limited availability of raw materials, restricted production capacity, remain pressing issues that need to be addressed. The integration of electrospinning with 3D printing enables the creation of complex multimaterial structures with complex physical and chemical properties.

Microfibrils are long, thin and flexible materials with large specific surface area and various mechanical properties, which can effectively realize material exchange and structural support, so they can be applied as carriers of cells or micro-tissues *in vitro*. It can also successfully assemble functional 3D structures by folding, bonding and weaving. As a new spinning method, microfluidic spinning technology is widely used to produce microfibers with designed structure or components owing to the precise control of liquid flow and the characteristics of laminar flow [199] (Figure 5C). This process is mild, rapid and does not influence the viability of encapsulated cells [200]. In recent years, researchers have carried out research on the use of micro cell carriers in repairing tissue defects, thus, avoiding the use of implants for tissue regeneration in large-scale surgery. Some studies have used microfluidic technology to prepare highly open porous microspheres, which provide a favorable microenvironment to facilitate the adhesion and proliferation of embedded myoblasts and enhance the cell differentiation. In addition, the results in mice confirmed the improvement of cell retention and vascularization as well as the differentiation of some muscle cells [201]. Grooved microfibrils with unique advantages can promote cell arrangement to simulate the microstructure of muscle. A microfluidic spinning system that can flexibly generate grooved microfibers by utilizing the volume change after cross-linking with different concentrations of sodium alginate has been proposed. The heterogeneous grooved microfibrils generated by GELMA and NAA were successfully applied as anisotropic scaffolds for 3D culture of C2C12 myoblasts. It was found that the myoblasts growing on the microfibrils maintained high viability and exhibited aligned organization, demonstrating that the heterogeneous fibers had favorable biocompatibility and topographical guidance capability [199]. The composition, structure and biocompatibility of microfibrils reveal the great potential of this method in skeletal muscle tissue regeneration.

The pore size of the microporous gel is adjusted by changing the freezing rate. For example, reducing the freezing rate will produce pores with larger diameter owing to the larger ice crystals [202]. This indicates that the scaffold morphology fabricated via freezing-based fabrication is predominantly governed by the structural attributes of ice crystals. Freeze drying technology not only provides excellent biocompatibility, high porosity and interconnectivity but also prevents the inactivation of proteins, enzymes and drugs at low temperature [203]. Directional freeze drying is a simple, economical and effective method for inducing arrangement. Directional freezing can obtain scaffolds with arranged porous or channel structures by controlling the solidification direction and the growth direction of ice crystals [180] (Figure 5D). In some studies, the synthesized conductive PPy nanoparticles were directly incorporated into the suspension of type I collagen and chondroitin sulfate, and then, freeze-drying was used to produce aligned and conductive 3D collagen scaffolds. The results showed that the conductivity of the scaffold increased by 5 times without adverse effects on the metabolic activity of myoblasts, and its aligned scaffold microstructure provided guidance clues to direct the growth of myoblasts and tissues, and promoted the formation and maturation of myotubes [178]. In some studies, GelMA was used as the matrix hydrogel, and silver nanowires (AgNW) as the conductive dopant, and the anisotropic conductive hydrogel scaffold for muscle defect repair was successfully prepared by directional freezing technology [173]. Although these synthesis technologies of scaffold have made some achievements in SMTE, researchers still have a long way to go to use the scaffolds produced by them in clinical research.

## Perspectives

Skeletal muscle possesses the capability to self-repair and maintain tissue integrity following minor injuries, primarily through the regeneration and repair processes, which aid in restoring the normal structure and function. However, in cases of severe defects or injuries, the intrinsic regenerative capacity of skeletal muscle might be insufficient to achieve complete structural and functional recovery. Under these circumstances, alternative and more effective therapeutic approaches become necessary. The clinical treatments, including surgical muscle flap transfers and physical therapy, have limitations, such as a restricted availability of donor tissue, associated surgical risks, and limited efficacy. SMTE represents an innovative approach that leverages tissue engineering techniques to repair and reconstruct severely damaged muscle tissue. As the technology continues to advance, SMTE demonstrates significant potential for enhancing repair and reconstruction of muscle tissue. Future research and optimization efforts are anticipated to provide more effective treatment options for a broader patient population.

In exploring the mechanism of self-repair and regeneration of skeletal muscle, we found that immune cells, especially macrophages, are pivotal to promote muscle regeneration, reduce inflammation and repair VML. By adjusting functional status, macrophages not only promote the clearance of necrotic tissue but also help the construction of new tissue after injury, which provides strong support for skeletal muscle regeneration. One of the major challenges in SMTE is to recognize the interplay between the immune system and the graft, mainly due to the complexity of different types of intercellular signaling cascades within the microenvironment. It also includes the dynamic balance and regulation of growth factors, cytokines and other bioactive molecules. This has inspired us to design a more refined and synergistic skeletal muscle regeneration strategy, combining inflammation regulation and immune response regulation with advanced biomaterials. Specifically, it means developing graft that can mimic the natural muscle microenvironment and promote the differentiation of macrophages toward tissue repair. At the same time, these materials need to be used as carriers to accurately deliver the necessary growth factors and regulatory factors to guide the behavior of immune cells and promote the proliferation and differentiation of muscle cells. As a double-edged sword for the treatment of VML, this strategy is not only expected to significantly improve the therapeutic effect of skeletal muscle injury but also may provide new inspiration and ideas for research in other tissue regeneration.

For SMTE, SCs were initially regarded as the best candidates. Once muscle is damaged, they can be activated and proliferate, differentiate into myotubes, and participate in the process of muscle repair and regeneration. However, they have the disadvantages of limited proliferation ability *in vitro*, high cost of enzymatic hydrolysis, cumbersome purification and low separation rate. Therefore, researchers have studied the potential of other myogenic cells, including MSCs, amniotic fluid stem cells, iPSCs, C2C12 cells, muscle SP cells in SMTE. Although all of the above cell types have shown certain application prospects, there is no consensus on which cell type is the most ideal source of muscle tissue regeneration. This is mainly because different cell types have their own advantages and disadvantages in terms of proliferation ability, differentiation efficiency, immunogenicity, safety and operability in practical applications. Therefore, further in-depth exploration of the molecular mechanisms of the seed cells and understanding their key signaling pathways regulating muscle regeneration and development are of great significance for optimizing cell culture conditions, improving the efficiency of cell therapy and promoting the development of SMTE.

Engineered biomaterials exhibiting tailored physicochemical characteristics have been characterized for their architectural and functional attributes. In these strategies, biomaterials can be used alone or in combination with cultured seed cells or exogenous growth factors, which are thought to be participants in endogenous repair. Notably, the application of conductive materials in muscle tissue engineering has opened up new prospects in recent years. These conductive materials not only transmit electrical signals and simulate electrophysiological activities in muscle tissue but also show unique advantages in promoting electrical coupling between muscle cells and enhancing muscle contraction function. The introduction of conductive materials enables biomaterial scaffolds not only have the dual functions of physical support and chemical induction but also can regulate cell behavior under electrical stimulation, which further improves the biological functionality and therapeutic effect of tissue engineering products. In addition, the porosity of skeletal muscle scaffolds is critical for myoblast infiltration and myofiber ingression, but it has not been fully characterized. The arrangement of scaffolds is also an important condition that needs to be met for skeletal muscle regeneration, to deliver mechanical forces efficiently. Therefore, a preparation method that can simultaneously adjust the pore size and establish the scaffold alignment is necessary. However, systematic quantification of these parameters is frequently absent in experimental protocols. Further research should explore the optimal pore size and arrangement of scaffolds fabricated using micro/nanotechnology and 3D printing methods.

The loss of nerve and vascular tissue can exacerbate injury of skeletal muscle and lead to atrophy, with vascularization and nerve regeneration being the main challenges in reconstructing skeletal muscle defects. Therefore, exploring and implementing a combined treatment strategy that addresses vascular tissue, neuromuscular junction and muscle regeneration is crucial. This strategy seeks to achieve functional synergy by concurrently promoting the reconstruction of them through a multidisciplinary approach. It necessitates a deep understanding of the biological properties and interactions among these components, as well as the development of innovative materials and treatments capable of precisely regulating these complex processes. Although our discussion is limited, new technologies continue to emerge, supported by advances in material chemistry and manufacturing techniques, and SMTE is expected to make great progress, with the expectation of developing effective strategies to meet effective strategies for regeneration of muscles, blood vessels and nerves. For instance, the design and innovation of biomaterials enable the construction of scaffolds with superior biocompatibility, degradability and specific induction functions, providing an optimal microenvironment for the growth, differentiation and integration of muscle, nerve and blood vessel cells. We highlight the need for vascularized 3D cultures combination with engineered muscle to sustain high metabolic demand, alongside mitochondrial enrichment strategies (e.g. PPAR-δ agonists) and nutrient-gradient biomaterials. Metabolic activity can be quantified via oxygen consumption rate (OCR) assays and metabolomic profiling. In addition, co-culture of motor neurons and myoblasts, the use of biomimetic scaffolds containing neurotrophic factors, and the application of dynamic electrical stimulation to simulate neural signal transduction may be effective measures to promote the formation and functional maturation of NMJ. Moreover, incorporating mechanoresponsive cells or biohybrid sensors into engineered constructs to detect and transmit mechanical stimuli may help in the recovery of proprioceptive function. Furthermore, the incorporation of technologies such as gene editing, cell therapy and 3D bioprinting enhances the precision and personalization of treatment strategies, offering robust technical support for their efficient regeneration.

Clinically, transplantation of myoblasts has been considered the most promising treatment, but xenogeneic sources face huge immune rejection [204]. Despite the autologous or stem cell sources avoid immune rejection, challenges remain in achieving effective cell engraftment and integration. This may be due to improper engraftment of transplanted cells and excessive dosage [205]. These challenges can be addressed through the utilization of biomaterials. While traditional scaffolds have demonstrated potential in promoting myogenic differentiation, they often lead to substantial fibrotic tissue formation *in vivo* and pose difficulties for clinical application [194, 206]. Clinically, neuromuscular electrical stimulation has been effective in increasing skeletal muscle strength and mobility in patients [207]. Graphene-family nanomaterials (GFNs) and carbon nanotubes (CNTS) show significant prospect in the field of electrical stimulation because of their unique physicochemical properties. However, safe conditions for the clinical application of these nanomaterials remain unclear [194]. Furthermore, translating these advancements to clinical applications requires overcoming anatomical barriers such as vascularization and neural connectivity [81]. In conclusion, although the development of SMTE holds promise, successful clinical translation requires an integrated approach that addresses compatibility, fibrosis, vascularization, reinnervation and other factors.

In conclusion, the ongoing integration and innovation in materials, bioengineering and medical technology hold promise for developing more efficient and safer strategies to address the clinical needs of muscle, vascular and nerve regeneration, thereby significantly advancing the field of skeletal muscle injury repair.
